# The Effectiveness of Intranasal Oxytocin on Addiction Severity Index and Anhedonia Symptoms in an Alcoholic Case with Oropharyngeal Cancer, a Protocol for a Single-case Experimental Design Pilot Study

**DOI:** 10.22037/ijpr.2020.14338.12314

**Published:** 2020

**Authors:** Bijan Pirnia, Mostafa Hamdieh, Maryam Kazemi Ashtiani, Parastoo Malekanmehr, Kambiz Pirnia, Alireza Zahiroddin, Paria Sadeghi

**Affiliations:** a *Department of Psychology, Faculty of Humanities, University of Science and Culture, Tehran, Iran. *; b *Cancer Research Center, Shahid Beheshti University of Medical Sciences, Tehran, Iran. *; c *Department of Psychosomatic, Taleghani Hospital, Faculty of Medicine, Shahid Beheshti University of Medical Sciences, Tehran, Iran. *; d *Department of Counselling, Faculty of Psychology, Arak Branch, Islamic Azad University, Arak, Iran. *; e *Department of Psychology, Faculty of Psychology, Tonekabon Branch, Islamic Azad University, Tonekabon, Iran. *; f *Bijan Center for Substance Abuse Treatment, Tehran, Iran. *; g *Department of Psychiatry, Behavioral Sciences Research Center, Shahid Beheshti University of Medical Sciences, Tehran, Iran* *.*; h *Department of Psychology, Tehran Science and Research Branch, Islamic Azad University, Tehran, Iran.*

**Keywords:** Oxytocin, Oropharyngeal Cancer, Alcoholism, Anhedonia

## Abstract

One of the goals of all pharmacological interventions aimed to increase the survival rate of patients with alcohol-dependent oropharyngeal cancers is to decrease alcohol use. Oxytocin is an alternative therapy for craving and alcohol management. However, the effectiveness of oxytocin on the severity of alcohol dependence has not been evaluated. In an ABABC study with a 6-month follow-up, during February 2015 to June 2016, a 67-year-old man with oropharyngeal squamous cell carcinoma with comorbidity of alcohol dependence syndrome and anhedonia was selected by Respondent-Driven sampling (RDS). The patient was treated with intranasal oxytocin in two six-week stages (B_1_ and B_2_) and received placebo only in the other two stages (A_1_ and A_2_), and the follow-up results were evaluated at stage C. The data were analyzed by Generalized Estimation Equation (GEE) and Repeated Measures Correlation (rmcorr). Primary outcomes showed that addiction severity Index (ASI) was significantly reduced in five domains of medical status, occupational status, alcohol consumption, family status, and mental status (all* p*’s < 0.05). There was no significant effect of treatment on legal status (all* p*’s > 0.05). Also, social (*p* < 0.05) and physical (*p* < 0.01) anhedonia syndrome decreased in the treatment stages. However, these changes did not persist until the 6-month follow-up (all* p*’s > 0.05). Secondary outcomes showed that there was a significant direct relationship between the severity of addiction and anhedonia (r_mcorr_ = 0.01). The findings of this study showed that the reduction of oxytocin-induced neurotoxic symptoms led to a decrease in the severity of addiction and an improvement in the anhedonia syndrome.

## Introduction

Oropharyngeal squamous cell carcinoma (OPSCC) is a rare disease that is affected by chronic alcohol consumption (1). Along with routine treatment of cancers, stopping, controlling or reducing alcohol consumption can increase survival rate in these patients.

 Despite the benefits of alcohol withdrawal, avoidance from using is associated with sleep disorders, restlessness, and depression syndrome, indicating chronic stress-driven activity (2). In addition, decreasing self-esteem in interpersonal interactions in these patients may lead to social deprivation and finally anhedonia symptoms.

Anhedonia or the inability to experience pleasure is a mood feature that is often seen in melancholic depression. It is also common in drug and alcohol users and is even cited as one of the main indices of alcohol use disorder (AUD) and it is due to a lack of dopaminergic mesolimbic activity during long periods of consumption or abstinence period (3).

Oxytocin, due to its potential in craving modulation, affects body signal transmission and has been suggested as a potential treatment for alcohol abuse disorders (4). Research evidence indicates the efficacy of oxytocin in promoting social interactions and improving psychological characteristics in alcohol-dependent individuals (5). The mechanism of action of oxytocin in the brain is the inhibitory effects of corticotropin-releasing factor on GABAergic interneurons in the amygdala and hypothalamus nuclei (2). Research findings indicate the relationship between continued alcohol consumption and decreased oxytocin levels in the brain that could be considered as a mechanism for changes in social behavior, stress response, and exacerbation of alcohol abuse disorder (6).

Previous studies have shown that oxytocin intranasal can reduce alcohol consumption by reducing neural Cue reactivity in brain networks (7). In clinical studies, oxytocin has also been shown to reduce the use of ethanol, cocaine, and methamphetamine (8).

Although studies on the efficacy of oxytocin in reducing alcohol use have been conducted, the efficacy of oxytocin on the severity of alcohol dependence and anhedonia syndrome has not been evaluated yet.

## Experimental

**Case Report**

We report a 67-year-old man with alcohol dependence disorder and anhedonia symptoms and oropharyngeal squamous cell carcinoma with bone and lung metastasis. To select the study sample, the respondent-driven sampling (RDS) method was used that is a combination of snowball or chain sampling and a mathematical model (Markov Chain Theory and Network Bias) and it is taken into consideration nowadays in large health organizations such as World Health Organization (9). The data for this study were collected during February 2015 and June 2016 (TCTR20180511003). 

Cancer Diagnosis: The patient was diagnosed with oropharyngeal squamous cell carcinoma two years prior to the present study (according to the union for international cancer control’s (UICC) criteria: Stage PT2, PN1) and eleven months after diagnosis, he underwent radical resection surgery. Alcoholism: The patient had heavy alcohol consumption for at least 20 years (consuming at least 24 standard drinks per week), and according to the international classification of diseases, ninth revision, clinical modification (ICD**-**11), he was diagnosed as an alcohol use disorder (ICD-code: 303). Classification of alcohol use disorders, although simplified in ICD-11, was in almost perfect agreement with the classifications of ICD-10 and DSM-IV (10). Also, the structured clinical interview for DSM-5 (SCID**-**5) was used to confirm the diagnosis of alcohol use disorder (Cohen’s kappa coefficient = 0.89). In study of Shankman *et al.* (11), the SCID’s severity scales demonstrated substantial internal consistency (all Cronbach’s αs >.80), test-retest reliability, and concurrent and predictive validity. 

Anhedonia Symptoms: According to the patient report, for the past 20 years there have been symptoms of anhedonia or a disability in the experience of pleasure and after the diagnosis of carcinoma, the severity of the symptoms was added to the diagnosis of dysthymia and after the SCID-5, the diagnosis of dysthymia was confirmed. Initiation of chemotherapy: Five months prior to the study, confirmation of bone and lung metastasis was confirmed through Computed Tomography (CT) scan, and the patient after seven weeks of concurrent chemotherapy with cisplatin, received three courses of combined chemotherapy with cisplatin and Fluorouracil (5-FU). Patient Care: The patient was a railroad retiree with a 36-year-old son and a 58-year-old sister who were primarily responsible for patient care and follow-up of medical and psychiatric treatments. According to the patient and his family, patient care standards have been met at an acceptable level. Psychological Support: Due to the interplay of the physical and psychological circles as well as the experience of depressive states associated with alcohol abuse and cancer treatments, the patient was deprived of a supportive social circle and most of the time was isolated. He spent limited hours with his son and sister.

Monitoring and Evaluating Alcohol Use: Alcohol consumption rate during the study process was assessed by self-report of the patient (Addiction Severity Index) and daily monitoring of his son from consumption (by dose). Psychiatric Drug Use: The patient was treated with oxytocin for one month before treatment with a 100 mg dose of venlafaxine early in the day before bedtime.

The intervention phases: We used a reversal A_1_B_1_A_2_B_2_C_1_ design with a multiple baseline and 6-month follow-up that A was the baseline stage and B was the intervention phase and C was considered as follow-up. The validity and accuracy of single-case experimental design have been confirmed in numerous studies (12, 13). The importance of using single recursive designs is due to having high internal validity through histrionic and maturation control and no need for independent control as much as it is called as “gold standard” in evaluation (14). Number of Visits: in baselines of A_1_ and A_2_ (four weeks, four evaluations) only evaluation was done and no intervention was performed. In phase B_1_ (six weeks, six evaluations) and B_2_ (six weeks, six evaluations), the patient was administered oxytocin intranasally. To reduce oxytocin plasma levels for evaluation as the second baseline, the washout period between two phases B_1_ and A_2_ was considered as 2 months. Also, the stability of the changes was evaluated in the form of a 6-month follow-up (C_1_, four weeks, four evaluations). The whole study period was 14 months and 24 evaluations were performed. Assessments continued by telephone during the washout period and the 6-month follow-up period. 

Oxytocin Treatment: Given that oxytocin is peptide, delivery from intranasal pathway is considered as a preferred method in clinical studies (15). Intranasal Oxytocin Spray (Syntocinon (®); Novartis, Basel, Switzerland) included oxytocin plus glycerol, sorbitol, benzyl alcohol and distilled water that was contained in an amber 7 mL glass nasal spray with a metered pump. The placebo sprays were similar in composition but did not contain oxytocin. Each pump spray delivered 50 μL of oxytocin (9 international unit (IU)) or placebo. All sprays were stored safely at cool temperature (4 °C). The patient was instructed to use the nasal spray correctly and to record doses in the notebook, and a timer was used to recall. Bottles were collected at the end to evaluate the total amount and dose estimates used. Medications, taken all days (except for the first day administered by the therapist) was administered by the patient himself in two meals before breakfast (about 10 a.m.) and before afternoon snack (about 3 p.m.) each time with an 18-IU dose (9 IU puff per each hole). SCAD-5, fifth edition of ASI, Revised Social Anhedonia Scale (RSAS) and Revised Physical Anhedonia Scale (RPAS) were used in this study (16, 17). Oxytocin Response: The response to oxytocin in the form of evaluation of addiction severity index and anhedonia was considered as the primary outcome and the relationship between the two indices was considered as the secondary outcome. All interviews were digitally recorded, transcribed and coded. All data in this study were collected after agreement with the patient and informed written consent was obtained prior to intervention and publication of the findings. All stages of the study were performed according to the latest version of the Declaration of Helsinki (DoH). The data were analyzed by generalized estimation equation test and repeated measures correlation in IBM SPSS Statistics V22.0.

**Figure 1:**
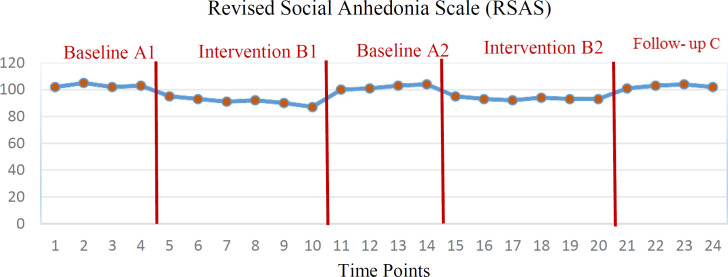
Distribution of Social Anhedonia scores during the 5 evaluation stages

**Figure 2:**
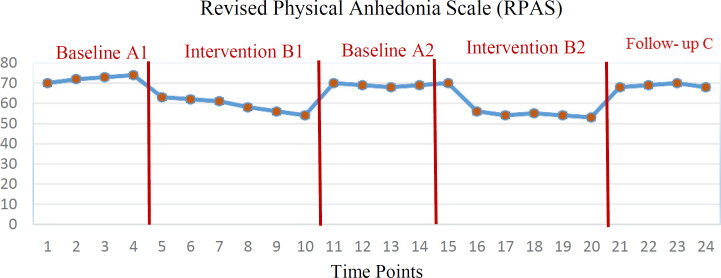
Distribution of physical Anhedonia scores during the 5 evaluation stages

Primary outcomes showed that 12 weeks of treatment in stages B_1_ and B_2_ had a significant effect on the five components of medical status, occupational status, alcohol use, family status, and mental status in the severity of addiction index (*all p’s* < 0.05). But the effectiveness of treatment on legal status was not significant (*all p’s* > 0.05). The findings also showed that intranasal oxytocin was effective on two social (Figure 1) and physical (Figure 2) anhedonia indices (*all p’s *< 0.01). However, the efficacy of treatment on both variables of addiction severity index and anhedonia did not persist until the follow-up phase (*all p’s *> 0.05). 

Secondary outcomes showed that there is a direct and significant relationship between addiction severity index and social and physical anhedonia (r_mcorr _= 0.01).

**r**_mcorr_ (1, Addiction Severity - Social Anhedonia) = 0.76, 99% CI [0.82, 0.60], *p* < 0.01

**r**_mcorr_ (1, Addiction Severity – Physical Anhedonia) = 0.71, 99% CI [0.71, 0.45], *p* < 0.01

## Discussion

Twelve weeks of oxytocin intranasal administration significantly reduced the five components (medical status, occupational status, alcohol use, family status, and mental status) of the six components of the addiction severity index and social and physical anhedonia syndrome. However, these changes were not maintained until follow-up stage. There was also a significant direct relationship between the severity of addiction and anhedonia.

In line with the findings of the present study, the results of the study by Faehrmann *et al.* (2) showed that oxytocin can reduce the mechanism of craving in alcohol-dependent individuals by reducing anxiety and depression and assist the patient in the abstinence phase. In terms of brain mechanisms, oxytocin reduces craving, cue reactivity, and recurrence by modulating the association between the Accumbens Nucleus and the cortical regions involved in craving for alcohol cue reactivity task (18). Contrary to our findings, the results of the study by Tunstall *et al.* showed that oxytocin increases the motivation for alcohol consumption in mice by affecting the GABAergic transporter (19).

Consistent with the findings of our study, the results of Mitchell *et al.* study showed that intranasal oxytocin has the potential to improve social perception and ultimately reduced alcohol consumption (20).

Part of the findings of the present study showed that oxytocin improved the patient’s mental and medical condition. Consistent with our findings, Afrisham *et al.* study showed that mental stress decreases oxytocin levels and cancer progression and that reducing stress via oxytocin can help improving medical condition in the patients (21).

Part of the findings of the present study showed that intranasal oxytocin was effective in reducing the symptoms of anhedonia. Consistent with our results, the findings of Love *et al.* demonstrated that oxytocin reduces symptoms of depression in alcohol-dependent patients by improving social interactions (5).

Secondary outcomes showed that there was a significant direct relationship between anhedonia syndrome and the severity of addiction to alcohol. The study by De Almeida Magalhães *et al.* demonstrated that ethanol consumption decreased the activity of the HPA axis, which could influence stress response with mediating of serotonergic system and affect mood indices (22). The results of a meta-analysis study showed that the relationship between treatment of alcohol dependence and the reduction of depression syndrome is a reciprocal one (23).

It seems that modulation of craving by inhibiting the effects of corticotropin on GABAergic interneurons can reduce the severity of addiction to alcohol, modulate stress response, and improve social behavior.

One of the limitations of this study was the use of self-report measures of depression and severity of addiction to alcohol that could be associated with bias. Also limited number of samples and presence of multiple intruding factors were important limitations of this study. It is recommended that future studies use biological assessments to investigate these variables. Running a randomized controlled trial on the efficacy of oxytocin in people with a diagnosis of alcohol use disorder can be a good rout for future studies.

## Conclusion

The findings of the present study were in line with research background suggesting the efficacy of intranasal oxytocin in reducing the severity of addiction and anhedonia syndrome in a patient with oropharyngeal squamous cell carcinoma with alcohol-dependent symptoms and anhedonia syndrome. A clinical trial to evaluate the efficacy of oxytocin in alcoholic patients is suggested. Investigating the role of depression severity in the effectiveness of oxytocin on reducing the severity of addiction can be a good rout for future studies.
